# Association among metabolic status, oxidative stress, milk yield, body condition score and reproductive cyclicity in dairy buffaloes

**DOI:** 10.1111/rda.14086

**Published:** 2022-01-29

**Authors:** Muhammad Najmus Saqib, Muhammad Subhan Qureshi, Syed Muhammad Suhail, Rifat Ullah Khan, Giancarlo Bozzo, Edmondo Ceci, Vito Laudadio, Vincenzo Tufarelli

**Affiliations:** ^1^ Department of Livestock, Breeding and Genetics Faculty of Animal Husbandry & Veterinary Sciences The University of Agriculture Peshawar Pakistan; ^2^ College of Veterinary Sciences Faculty of Animal Husbandry & Veterinary Sciences The University of Agriculture Peshawar Pakistan; ^3^ Department of Veterinary Medicine University of Bari “Aldo Moro” Bari Italy; ^4^ Section of Veterinary Science and Animal Production Department of DETO University of Bari “Aldo Moro” Bari Italy

**Keywords:** buffaloes, metabolites, milk production, oxidative stress, progesterone

## Abstract

The aim of this study was to determine the metabolic status, stress and ovarian activity through progesterone profile in dairy buffalo as influenced by post‐partum days, body condition score (BCS) and lactation number. A total of 45 dairy buffaloes were involved and divided into three factors based on their BCS (2.5, 3.0 and 3.5, respectively), lactation number (1, 2 and 3, respectively), and post‐partum intervals (14, 28, 42 and 56, respectively). Based on findings, from day 14 to day 56 after parturition, blood glucose, cholesterol and triglyceride levels increased significantly (*p* < .01), while malondialdehyde (MDA) and cortisol levels decreased significantly (*p* < .05) on day 56 of the trial. With increased BCS levels, milk yield, blood metabolites and progesterone improved significantly (*p* < .05), whereas stress indicators decreased significantly (*p* < .01). Blood metabolites, progesterone and milk production were significantly (*p* < .01) higher and stress indicators (MDA and cortisol) were significantly (*p* < .01) lower in lactation stage. Buffaloes with a greater lactation stage and growing post‐partum stage had better blood metabolite and progesterone concentrations and less stress. It was concluded that better BCS and increased lactation stage have significant impact on milk yield, stress reduction and restoration of ovarian activity in buffaloes during post‐partum period.

## 1 INTRODUCTION

The transition period, which lasts 21 days before and after parturition, is the most essential period for dairy animals. Dairy cows are sensitive to metabolic problems and oxidative stress during the physiological phases of pregnancy and lactation (Ullah et al., 2020). The peri‐parturient phase has a significant impact on the health and subsequent performance of dairy animals since they undergo significant metabolic and physiological changes during this time (Alharthi et al., 2021; Piccione et al., 2012). Negative energy balance (NEB) during early lactation is one of the common physiological causes of oxidative stress and health concerns in dairy animals (Elsayed et al., 2019; Saqib et al., 2018; Zhao et al., 2019). Dairy cows adjust to multiple hormonal and metabolic changes associated with the end of pregnancy and the start of lactation during the transition period (Colakoglu et al., 2019). Early production and NEB exacerbate the production of fatty acids due to body fat mobilization resulting in frequent changes in body condition score (BCS). The high‐yielding dairy cow faces a major metabolic strain during the transition from late pregnancy to early lactation, and haematochemical profiles are critical in assessing the health state of animals during this time (Ihsanullah et al., 2017; Piccione et al., 2012; Saqib et al., 2018; Ullah et al., 2019b).

The BCS is a quick and easy way to analyse an animal's energy reserves by measuring fat metabolism and its relationship to energy metabolism (Hoedemaker et al., 2009; Shah et al., 2019; Ullah et al., 2019a). Since BCS is related to the fertility status of dairy cows (Laubenthal et al., 2017). Cows having a greater BCS at calving exhibited higher fatty acid concentrations in early lactation than cows with a lower BCS (Arfuso et al., 2016). Oxidative stress is more responsive in cows with a higher BCS (Alhidary et al., 2016a,2016b; Saqib et al., 2018). Oxidative stress is caused by an imbalance between reactive oxygen metabolites and antioxidants (Ullah et al., 2019b) and various oxidative status markers such as malondialdehyde (MDA) were found to be greater in cows with higher BCS before calving. It is well known that blood metabolites and BCS levels in dairy animals substantially change during early lactation. These modifications could reveal crucial details regarding reproduction and herd management (Bernabucci et al., 2005; Kim & Suh, 2003). Pre‐partum BCS and its effects on post‐partum blood metabolites, oxidative stress, fertility and milk production have gotten very little attention in dairy buffaloes. The hypothesis of the present research was how the metabolic, stress and ovarian activity via progesterone fluctuation are affected by post‐partum period, BCS and lactation stage.

Therefore, the present study was designed to evaluate the impact of perceiving BCS and lactation numbers on metabolic status, milk yield, stress indicators and resumption of ovarian activity through progesterone profile in dairy buffaloes during the post‐partum period.

## 2 MATERIALS AND METHODS

The study was approved by the Departmental Committee on Ethics and Animal Welfare, Faculty of Animal Husbandry and Veterinary Sciences, The University of Agriculture, Peshawar (Approval no. 78/LM/UAP/20).

### 2.1 Selection of animals

A total of 45 multiparous dairy buffaloes (5–7 years) were involved and divided into three groups based on BCS (2–2.5, 2.5–3.0 and 3.0–3.5), lactation stage (1, 2 and 3), and post‐partum days (14, 28, 42, and 56) 21 days after calving. The buffaloes were fed on cut‐and‐carry fodder system that included seasonal green fodder, wheat straw, and cotton seed cakes, covering their nutrients requirements as recommended by NRC (1978). Water was available *ad libitum*. The ingredients and nutrients composition are given in Table 1. Nutritive values of the ingredients were analysed with AOAC (1990). The buffaloes were kept in open paddock under similar conditions of natural photoperiod and temperature. Buffaloes were sampled for blood, faeces and skin scraping technique to ensure that the animals were free of external and internal parasites. Buffaloes were milked twice manually.

**TABLE 1:** Ingredients and nutritive value (%) of diet fed to dairy buffaloes

Ingredients	Inclusion level (%)	DM	CP	CF	Ash	TDN
Cotton seed cake	17	89.63	19.51	25.89	09.32	63.47
Commercial concentrate	10	90.51	16.73	14.63	06.54	74.56
Wheat bran	13	84.32	14.56	08.21	05.71	70.44
Wheat straw	15	91.45	07.44	43.54	12.45	40.73
Maize stover	20	86.77	05.76	45.37	10.36	51.37
Berseem	25	14.36	13.22	19.51	14.53	66.51

Abbreviations: CF, crude fibre; CP, crude protein; DM, dry matter; TDN, total digestible nutrients.

During the sampling period, the physiological parameters (BCS, general health status and milk yield) of the buffaloes were recorded. The BCS was calculated using the score method outlined by Peters and Ball (1987) briefly described as: score 2.0–2.5 – weak; when round transverse processes were observed and palpated with thin fat covering; score 2.6–3.0 – normal; when firm pressure was needed to feel individual transverse processes due to good muscular and fat covering; score 3.1–3.5 – slightly fat; when transverse vertebral processes could not be felt during palpation with firm pressure.

Health status was evaluated with general characteristics of healthy animal, which behaves normally and stays with herd, bright eyes and moist muzzle without discharge from nose no wounds and eating normally. Visiting veterinary practitioners, fellow farmers, and other services providers helped to build farming techniques based on communicative expertise and some engagement. Buffaloes were clinically examined weekly after parturition for rectal temperature, heart rate and respiration rate, external and internal genitalia (data not shown).

### 2.2 Blood and milk sampling and analysis

During the diestrous period, blood samples were taken on post‐partum days 14, 28, 42 and 56, respectively. Early in the morning before feeding, the blood samples were drawn from the jugular vein of dairy buffalos with a disposable syringe. The blood sample was poured into a vacutainer glass tube without anticoagulants, labelled, placed in a chilled container and sent to the laboratory for analysis. Serum was decanted (30 min) through centrifuge machine at 1000 *g* × 15 min and stored at −20°C until utilized. Spectrophotometer and commercially available kits (Biotech Reagent Biotechnical Co. Ltd.) were used to quantify oxidative stress marker, such as malondialdehyde (MDA) and some other blood metabolites (triglycerides, glucose and cholesterol). Serum cortisol was determined using commercial kit with the help of ELISA reader. With the use of an ELISA reader (Anthos 2000, Anthos Labtech Instruments, and Wals Salzburg), serum cortisol and progesterone (fertility marker) were determined using commercial kits (Biosource Europe S.A. Rue de Industry 8, 1400 Nivelles).

Daily milk yield was measured by emptying cow udder completely through gentle hand milking early in morning while practicing same procedure at evening at day 14, 28, 42 and 56, respectively. Combining the weight of both times milking were used to calculate the daily milk yield.

### 2.3 Statistical analysis

Data were put into MS Excel‐2007 and analysed through SPSS (2004) with the help of general linear model using repeated measures. Interaction was used between lactation and post‐partum days. Mean of different parameters were compared using Turkey's test. Pearson's correlation coefficient was determined for various interactions. Moreover, percent change for each independent variable was calculated as the percent change between the highest and lowest values recorded.

## 3 RESULTS

Table 2 shows the mean values of the examined parameters at various post‐partum days. This table reveals that post‐partum intervals have a statistically significant impact on blood glucose, cholesterol, triglycerides, progesterone and cortisol levels (*p* < .01). From day 14 to day 56 after parturition, blood glucose, cholesterol and triglyceride levels increased, while MDA and cortisol levels decreased. Table 3 presents the effect of BCS on changes in blood metabolites, milk yield and stress markers. With increased BCS levels, milk yield, blood metabolites and progesterone levels improved, whereas stress signs decreased. The MDA is indicative of oxidative stress in animals, while cortisol represents the overall stress. Both are declining as the BCS rises from 2.5 to 3.5. Table 4 reports changes in various parameters with the increasing lactation number. Blood metabolites, milk yield and progesterone level were enhanced (*p* < .01) with increasing lactation number. Blood metabolites, progesterone and milk production were significantly (*p* < .01) higher in lactation number 3. Stress indicators (MDA and cortisol) were significantly (*p* < .01) lower in lactation number 3.

**TABLE 2:** Metabolic, stress and reproductive indicators of buffaloes on different post‐partum days

Post‐partum days	Metabolic indicators			Stress indicators		Reproductive indicator
	Glucose (mg/dl)	Cholesterol (mg/dl)	Triglyceride (mg/dl)	MDA (µmol/l)	Cortisol (µg/dl)	Progesterone (ng/ml)
14	47.21^d^	133.65^d^	26.51^d^	21.32^a^	18.14^a^	2.04^d^
28	52.06^c^	138.29^c^	30.46^c^	19.46^b^	17.77^ab^	3.13^bc^
42	56.94^b^	142.69^b^	34.74^b^	17.05^c^	16.03^c^	3.45^ab^
56	62.22^a^	147.08^a^	38.86^a^	15.73^d^	15.51^d^	3.79^a^
Change (%)	+ 24.76	+ 9.13	+ 12.35	− 26.21	− 14.49	+ 46.17
Pooled *SEM*	0.7	0.54	0.61	0.44	0.76	0.01

Note

Mean values with different superscripts within the column differ significantly (*p* < .01); Change %: is the percent change between the highest and lowest values in a column.

**TABLE 3:** Metabolic, productive and reproductive indicators of buffaloes under different BCS

BCS	Metabolic indicators			Production	Stress indicators		Reproductive indicator
	Glucose (mg/dl)	Cholesterol (mg/dl)	Triglyceride (mg/dl)	DMY (l/head)	MDA (µmol/l)	Cortisol (µg/dl)	Progesterone (ng/ml)
2.5	45.07^c^	133.53^c^	25.29^c^	3.13^c^	19.45^a^	21.80^a^	2.07^c^
3.0	51.95^b^	139.25^b^	28.17^b^	4.37^b^	15.67^b^	17.02^b^	3.04^ab^
3.5	62.93^a^	145.09^a^	32.05^a^	5.21^a^	11.72^c^	13.05^c^	3.55^a^
Change (%)	+ 28.38	+ 7.96	+ 21.09	+ 66.45	− 39.74	− 40.13	+ 44.69
Pooled *SEM*	0.32	0.32	0.11	0.21	0.56	0.78	0.01

Note

Mean values with different superscripts within the column differ significantly (*p* < .01); Change %: is the percent change between the highest and lowest values in a column.

Abbreviations: DMY, daily milk yield; MDA, malondialdehyde.

**TABLE 4:** Metabolic, productive and reproductive indicators of buffaloes under different lactations number

Lact No.	Metabolic indicators			Production	Stress indicators		Reproductive indicator
	Glucose (mg/dl)	Cholesterol (mg/dl)	Triglyceride (mg/dl)	DMY (l/head)	MDA (µmol/l)	Cortisol (µg/dl)	Progesterone (ng/ml)
1	49.98^c^	127.29^c^	29.78^c^	3.67^c^	24.50^a^	22.29^a^	2.15^c^
2	53.54^b^	129.84^b^	32.19^b^	4.52^b^	19.58^b^	16.74^b^	3.21^b^
3	59.29^a^	134.16^a^	35.97^a^	5.64^a^	13.70^c^	11.25^c^	3.73^a^
Change (%)	+ 15.70	+ 5.12	+ 17.20	+ 53.67	− 44.08	− 49.52	+ 42.35
Pooled *SEM*	0.76	0.68	0.71	0.45	0.22	0.32	0.01

Note

Means with different superscripts within the column differ significantly (*p* < .01); Change %: is the percent change between the highest and lowest values in a column.

Abbreviations: DMY, daily milk yield; Lact, lactation number; MDA, malondialdehyde.

Table 5 shows the interaction between post‐partum days and lactation number affecting the blood metabolites, stress and reproductive indicator. Although there was no significant (*p* > .05) interaction between these indicators, the animals with a greater lactation number and growing post‐partum stage had better blood metabolite and progesterone concentrations, as well as less stress, during the post‐partum phase. Moreover, interaction between BCS and lactation number were not significant (*p* = .076). Similarly, the interaction between BCS and post‐partum days was also not significant (0.065) (data not shown).

**TABLE 5:** Metabolic, stress and reproductive indicators of buffaloes as affected by lactation number and post‐partum days

Lactation	Post‐partum days	Metabolic indicators			Stress indicators		Reproductive indicator
		Glucose (mg/dl)	Cholesterol (mg/dl)	Triglycerides (mg/dl)	MDA (µmol/l)	Cortisol (µg/dl)	Progesterone (ng/ml)
1	14	37.33	120.63	23.46	27.34	23.12	1.09
	28	41.48	125.33	27.52	25.45	22.64	2.15
	42	43.52	129.47	31.93	23.56	21.95	2.33
	56	51.61	133.72	36.20	21.67	21.45	3.06
2	14	40.05	123.23	26.27	23.75	17.49	2.05
	28	43.01	127.77	30.00	20.86	17.16	3.01
	42	46.88	132.00	34.36	19.31	16.40	3.25
	56	54.24	136.35	38.12	17.43	15.91	3.44
3	14	45.24	127.09	29.80	15.55	13.81	2.41
	28	48.69	131.76	33.87	14.63	12.50	2.73
	42	53.43	136.60	37.95	13.35	11.72	3.33
	56	59.80	141.18	42.26	11.28	11.17	3.81
Pooled *SEM*		0.93	0.81	0.87	0.41	1.22	0.01
*p*‐values							
Lact		.321	.433	.062	.124	.441	.563
PP		.096	.870	.076	.651	.324	.122
Lact ×PP		.065	.123	.065	.322	.982	.434

Abbreviations: DMY, daily milk yield; Lact, lactation number; MDA, malondialdehyde; PP, post‐partum days.

Table 6 indicates the correlations among various blood metabolites, hormones and stress indicators. Cortisol was significantly negatively correlated with BCS and positively correlated with cholesterol. MDA was significantly negatively correlated with BCS and positively correlated with cortisol. Milk yield was positively correlated with BCS, cholesterol and glucose while negatively correlated with MDA. Progesterone concentration was positively correlated with BCS, cholesterol, glucose and negatively correlated with MDA and milk yield.

**TABLE 6:** Correlation among metabolic, stress, productive and reproductive indicators in dairy buffaloes during post‐partum period (14–56 days)

Parameters	BCS	Cholesterol	Cortisol	MDA	Glucose	DMY	Progesterone
Cholesterol	0.20 (0.02)	‐	‐	‐	‐	‐	‐
Cortisol	−0.04 (0.01)	0.47 (0.01)	‐	‐	‐	‐	‐
MDA	−0.36 (0.04)	−0.45 (0.01)	0.65 (0.02)	‐	‐	‐	‐
Glucose	0.22 (0.01)	0.97 (0.02)	−0.48 (0.01)	−0.76 (0.01)	‐	‐	‐
DMY	0.40 (0.02)	0.20 (0.03)	−0.34 (0.03)	−0.54 (0.02)	0.22 (0.05)	‐	‐
Progesterone	0.10 (0.03)	0.71 (0.04)	−0.81 (0.03)	−0.63 (0.02)	0.73 (0.04)	−0.91 (0.05)	‐
Triglyceride	0.25 (0.01)	0.96 (0.01)	−0.45 (0.02)	−0.34 (0.03)	0.95 (0.02)	0.51 (0.05)	0.69 (0.05)

Note

Values in brackets represent the *p*‐values.

Abbreviations: DMY, daily milk yield; MDA, malondialdehyde.

## 4 DISCUSSION

In the available published literature, studies have mostly been published in dairy cows during post‐partum period. Little attention has been given to the productive and reproductive performance in dairy buffaloes during post‐partum period. The objective of the present study was to understand the impact of pre‐partum BCS and lactation numbers on metabolic attributes, milk yield, stress indicators and resumption of ovarian activity in dairy buffaloes during post‐partum period. Changes in biochemical indices occur as a result of increased metabolic demands during both pregnancy and lactation, despite haemostatic systems that function to maintain blood parameters within physiological levels (Piccione et al., 2012). The need for the regulatory mechanism, responsible for the process involved in milking increases throughout the peri‐partum period. As a result, most animals experience distinct alterations in lipid metabolism during pregnancy and lactation (Roche et al., 2009).

In the current study, the levels of blood metabolites increased significantly as the number of post‐partum days grew from 14 to 56, resulting in higher blood levels of glucose, cholesterol and triglycerides and lower levels of stress markers (MDA and cortisol) during the phase of uterine involution. It reflects the recovery of the recently parturited buffaloes (resumption of ovarian activity) from parturition stress as mentioned through progesterone profile (Khan et al., 2016). The dietary quality, management, BCS, parity, lactation number and climatic conditions affect the post‐partum blood metabolites and stress indicators (Ciaramella et al., 2005; Habib et al., 2007; Khan et al., 2016). During the post‐partum period, dairy buffaloes go through a lot of physiological and hormonal changes. Negative energy balance is easily developed in the early post‐partum stage due to metabolic stress induced by a fall in dry matter intake and increased energy demands at calving and the start of lactation (Mishra et al., 2007). In the current study, glucose concentrations were shown to be greater in animals with a good body condition score. The concentration of glucose rises dramatically as the number of post‐partum days increased. Several researchers have supported the idea that blood glucose concentration indicates an animal's energy level (Dokovic et al., 2012; Kumar et al., 2010). Previous research reports (Hagawane et al., 2009; Mandali et al., 2002) found a direct link between glucose availability, production and reproductive activities in lactating animals throughout the post‐partum period. During the post‐partum period, animals with a satisfactory body condition score had stable blood metabolites levels, with the mammary gland using around 80% of the circulating blood metabolites for milk synthesis (Borghese and Moioli, 2011).

Serum cholesterol in the current study increased significantly as the number of post‐partum days increased. Cholesterol is a precursor to the steroid hormones, and an optimal level of blood cholesterol is essential to regulate physiological activities. Animals having good body condition score showed increased cholesterol level. The current findings were consistent with the previous studies reporting a higher level of blood cholesterol during the post‐partum period (Hagawane et al., 2009). In the present study, serum triglyceride level significantly increased with increasing post‐partum days. Triglyceride concentrations were high in animals having good body reserves (Piccione et al., 2012). Moreover, triglycerides concentrations represent good physiological conditions of the dairy animals (Zhao et al., 2019). The voluntary feed intake and proper nutrition has been strongly associated with increase in triglycerides level as reported in earlier studies (Bertics et al., 1992; Marai et al., 2004). Higher serum triglycerides concentration is associated with increased milk production (Ashmawy, 2015; Civelek et al., 2011). The increase in metabolic indicators with the increasing post‐partum period indicates the melting of the stored energy and increase energy demand during post‐partum period. Secondly, with the increasing post‐partum days, the animals are relieved of the parturition stress probably at the cost of the flow of the stored energy. These two factors assist the dairy buffaloes to reach the level of conception in the next phase as indicated from the progesterone level.

The stress indicators (MDA and cortisol) decreased when the BCS increased from 2.5 to 3.5 in this study. MDA signifies oxidative stress in animals, while cortisol represents the overall stress (Sampath et al., 2004). The imbalance between reactive oxygen species generation and antioxidant availability is referred to as oxidative stress (Majid et al., 2015; Rahman et al., 2014). This decrease in stress is most likely linked to an increase in blood progesterone levels. In this study, greater cortisol levels were linked to lower progesterone levels and vice versa. As a result, our findings support the alternate functions of the hypothalamus–pituitary–adrenal axis and hypothalamus–pituitary–gonadal axis. Because farmers are interested in milk yield, and farm‐born lactating cows of a younger age are preferred as lactating cows, the performance of the animals improves during the maturity phase as various body organs and systems develop, as evidenced by our blood metabolites and progesterone levels, as well as a decrease in stress indicators. The findings of this study are consistent with Mishra et al. (2007), who found a steady decrease in serum cortisol levels as post‐partum days increased. In the present study, the animals having good body condition score showed better performance. Blood metabolites and progesterone level improved, and stress indicators decreased with the increasing BCS levels. This enhancing stress level is associated with decreasing level of blood progesterone, indicating that hypothalamus–pituitary–adrenal axis and hypothalamus–pituitary–gonadal axis works alternatively (Roche et al., 2009). It is evident from the findings of the present study that animals with BCS 3.5 had better metabolic, productive and reproductive as well as stress level. It indicates that the animals with higher BCS prepare the buffaloes to better fight parturition and milk yield stress and prepare them earlier to resume reproductive cyclicity. Similarly, the dairy buffaloes in the higher rank of lactation had better metabolic, productive and reproductive performance with lower level of stress. It indicates the adaptability of the animals to the challenges of post‐partum metabolic, productive and reproductive and stress management demands.

In the present study, correlation of different parameters showed that milk yield was positively correlated with BCS and negatively correlated with cortisol and MDA. Similarly, progesterone was positively correlated with BCS and negatively correlated with cortisol, MDA and milk yield. The MDA is negatively correlated with BCS and positively correlated with cortisol. These findings indicated that for higher milk production, decreased level of MDA and cortisol is very important. Likewise, higher BCS is related to higher milk production and higher level of progesterone.

## 5 CONCLUSIONS

It was concluded that in dairy buffaloes, BCS, post‐partum days and lactation numbers have a substantial impact on blood metabolites, milk production, MDA, cortisol and progesterone. From day 14 to 56 after parturition, there was an increase in blood metabolites and a drop in stress markers, indicating that reproductive activity through progesterone profile was restored. With an increase in BCS from 2.5 to 3.5, blood metabolites, milk yield and progesterone levels increased showing a positive impact on dairy buffaloes. These findings indicate that metabolic and stress indicators as well as production and reproduction are greatly affected by post‐partum days, BCS ranking and lactation stage, hence the hypothesis was proved.

## CONFLICT OF INTEREST

None of the authors have any conflict of interest to declare.

## AUTHOR CONTRIBUTIONS

Conceptualization: M.N.S., M.S.Q. and S.M.S. Methodology: N.C. Validation: M.N.S. and R.U.K. Formal analysis: M.I. Investigation: M.N.S. and M.S.Q. Data curation: R.U.K. and V.T. Writing original draft preparation: M.N.S. and R.U.K. Writing review and editing: R.U.K., G.B., E.C., V.T. and V.L.

## Data Availability

Data that support the findings of this study are available upon request from the corresponding author.

## References

[rda14086-bib-0001] Alharthi, A. S. , Alobre, M. M. , Abdelrahman, M. M. , Al‐Baadani, H. H. , Swelum, A. A. , Khan, R. U. , & Alhidary, I. A. (2021). The Effects of different levels of sunflower hulls on reproductive performance of yearly ewes fed with pelleted complete diets. Agriculture, 11, 959. 10.3390/agriculture11100959

[rda14086-bib-0002] Alhidary, I. A. , Abdelrahman, M. M. , & Khan, R. U. (2016). Comparative effects of direct‐fed microbial alone or with a traces mineral supplement on the productive performance, blood metabolites and antioxidant status of grazing Awassi lambs. Environmental Science and Pollution Research, 23, 25218–25223. 27687757 10.1007/s11356-016-7684-z

[rda14086-bib-0003] Alhidary, I. A. , Abdelrahman, M. M. , Uallh Khan, R. , & Harron, R. M. (2016). Antioxidant status and immune responses of growing camels supplemented a long‐acting multi‐trace minerals rumen bolus. Italian Journal of Animal Science, 15, 343–349. 10.1080/1828051X.2016.1186502

[rda14086-bib-0004] AOAC (1990). Official methods of analysis, 16th ed. Association of Official Analytical Chemists.

[rda14086-bib-0005] Arfuso, F. , Fazio, F. , Levanti, M. , Rizzo, M. , Di Pietro, S. , Giudice, E. , & Piccione, G. (2016). Lipid and lipoprotein profile changes in dairy cows in response to late pregnancy and the early postpartum period. Archives Animal Breeding, 59(4), 429–434.

[rda14086-bib-0006] Ashmawy, N. A. (2015). Blood metabolic profile and certain hormones concentrations in Egyptian buffalo during different physiological states. Asian Journal of Animal and Veterinary Advances, 10(6), 271–280. 10.3923/ajava.2015.271.280

[rda14086-bib-0007] Bernabucci, U. , Ronchi, B. , Lacetera, N. , & Nardone, A. (2005). Influence of body condition score on relationships between metabolic status and oxidative stress in periparturient dairy cows. Journal of Dairy Science, 88, 2017–2026. 10.3168/jds.S0022-0302(05)72878-2 15905432

[rda14086-bib-0008] Bertics, S. J. , Grummer, R. R. , Cadorniga‐Valino, C. , & Stoddard, E. E. (1992). Effect of prepartum dry matter intake on liver triglyceride concentration and early lactation. Journal of Dairy Science, 75(7), 1914–1922. 10.3168/jds.S0022-0302(92)77951-X 1500587

[rda14086-bib-0009] Borghese, A. , & Moioli, B. (2011). Husbandry of dairy animals (Buffalo). Mediterranean region. In W. F. John (Ed.), Encyclopedia of dairy sciences. 780‐14 784.

[rda14086-bib-0010] Ciaramella, P. , Corona, M. , Ambrosio, R. , Consalvo, F. , & Persechino, A. (2005). Haematological profile on non‐lactating Mediterranean buffaloes (*Bubalus bubalis*) ranging in age from 24 months to 14 years. Research in Veterinary Science, 79, 77–80. 10.1016/j.rvsc.2004.11.004 15894028

[rda14086-bib-0011] Civelek, T. , Aydin, I. , Cingi, C. C. , Yilmaz, O. , & Kabu, M. (2011). Evaluation of serum nonesterified fatty acids and beta‐hydroxybutyrate in dairy animals with retained placenta. Pakistan Veterinary Journal, 31, 341–344.

[rda14086-bib-0012] Çolakoğlu, H. E. , Yazlık, M. O. , Pekcan, M. , Kaya, U. , Kaçar, C. , Vural, M. R. , Kurt, S. , Yildirim, M. M. , Bas, A. , & Küplülü, Ş. (2019). Impact of prepartum body condition score loss on metabolic status during the transition period and subsequent fertility in Brown Swiss dairy cows. Journal of Veterinary Research, 63(3), 375. 10.2478/jvetres-2019-0039 31572818 PMC6749747

[rda14086-bib-0013] Djokovic, R. , Samanc, H. , Petrovic, M. D. , Ilic, Z. , & Kurcubic, V. (2012). Relationship among blood metabolites and lipid content in the liver in transitional dairy cows. Biotechnology in Animal Husbandry, 28(4), 705–714. 10.2298/BAH1204705D

[rda14086-bib-0014] Elsayed, D. H. , Abdelrazek, H. M. A. , El Nabtiti, A. A. S. , Mahmoud, Y. K. , & Abd El‐Hameed, N. E. (2019). Associations between metabolic profiles, post‐partum delayed resumption of ovarian function and reproductive performance in Egyptian buffalo: Roles of IGF‐1 and antioxidants. Animal Reproduction Science, 208, 106134. 10.1016/j.anireprosci.2019.106134 31405461

[rda14086-bib-0015] Habib, G. , Hameed, A. , & Akmal, M. (2007). Current feeding management of peri‐urban dairy buffaloes and scope for improvement. Pakistan Veterinary Journal, 27(1), 35–41.

[rda14086-bib-0016] Hagawane, S. D. , Shinde, S. B. , & Rajguru, D. N. (2009). Haematological and blood biochemical profile in lactating buffaloes in and around Parbhani city. Veterinary World, 2, 467–469.

[rda14086-bib-0017] Hoedemaker, M. , Prange, D. , & Gundelach, Y. (2009). Body condition change ante‐ and postpartum, health and reproductive performance in German Holstein cows. Reproduction in Domestic Animals, 44, 167–173. 10.1111/j.1439-0531.2007.00992.x 18564311

[rda14086-bib-0018] Ihsanullah, M. S. , Qureshi, S. M. , Suhail, S. A. , & Khan, R. U. (2017). Postpartum endocrine activities, metabolic attributes and milk yield are influenced by thermal stress in crossbred dairy cows. International Journal of Biometeorology, 61, 1561–1569. 10.1007/s00484-017-1335-z 28393266

[rda14086-bib-0019] Khan, I. , Qureshi, M. S. , Akhtar, S. , Ali, I. , & Ullah, G. (2016). Fertility improvement in cross‐bred dairy cows through supplementation of vitamin e as antioxidant. Pakistan Journal of Zoology, 48(4), 923–930.

[rda14086-bib-0020] Kim, I. H. , & Suh, G. H. (2003). Effect of the amount of body condition loss from the dry to near calving periods on the subsequent body condition change, occurrence of postpartum diseases, metabolic parameters and reproductive performance in Holstein dairy cows. Theriogenology, 60, 1445–1456. 10.1016/S0093-691X(03)00135-3 14519466

[rda14086-bib-0021] Kumar, S. , Ataullah, S. , & Ramsagar, R. (2010). Comparative studies on metabolic profile of anestrous and normal cyclic Murrah buffaloes, Buffalo Bulletin, 29(1), 7‐11. Faculty of Veterinary Science and Animal Husbandry, R.S. Pura, Jammu, India.

[rda14086-bib-0022] Laubenthal, L. , Ruda, L. , Sultana, N. , Winkler, J. , Rehage, J. , Meyer, U. , Dänicke, S. , Sauerwein, H. , & Häussler, S. (2017). Effect of increasing body condition on oxidative stress and mitochondrial biogenesis in subcutaneous adipose tissue depot of nonlactating dairy cows. Journal of Dairy Science, 100, 4976–4986. 10.3168/jds.2016-12356 28342615

[rda14086-bib-0023] Majid, A. , Qureshi, M. S. , & Khan, R. U. (2015). *In vivo* adverse effects of alpha‐tocopherol on the semen quality of male bucks. Journal of Animal Physiology and Animal Nutrition, 99, 841–846. 25580862 10.1111/jpn.12284

[rda14086-bib-0024] Mandali, G. C. , Patel, P. R. , Dhami, A. J. , Rawal, S. K. , & Christi, K. S. (2002). Biochemical profile in buffaloes with periparturient reproductive and metabolic disorders. Indian Journal of. Animal Reproduction, 23(2), 130–134. 223.

[rda14086-bib-0025] Marai, I. F. M. , El‐Darawany, A. A. , Fadiel, A. , & Hafez, M. A. M. (2004). Reproductive traits and the physiology background of the seasonal variations in Egyptian Suffolk ewes under the conditions of Egypt. Animals of Arid Zone, 42, 1–9.

[rda14086-bib-0026] Mishra, A. , Pankaj, P. K. , Roy, B. , Sharma, I. J. , & Prakash, B. S. (2007). Antigonadotrophic effects of prolactin in relation to commencement of cyclicity, milk yield and blood metabolites in postpartum lactating riverine buffaloes (*Bubalus Bubalis*). Buffalo Bulletin, 26(3), 77–86.

[rda14086-bib-0027] NRC (1978). Nutrient requirements of dairy cattle. National Research Council: National Academy Press.

[rda14086-bib-0028] Peters, A. R. , & Ball, P. J. H. (1987). Reproduction in cattle (pp. 167–168). Butter worth’s.

[rda14086-bib-0029] Piccione, G. , Messina, V. , Marafioti, S. , Casella, S. , Giannetto, C. , & Fazio, F. (2012). Changes of some haematochemical parameters in dairy cows during late gestation, post partum, lactation and dry periods. Vet Med Zoot, 58(1), 59–64.

[rda14086-bib-0030] Rahman, H. , Qureshi, M. S. , & Khan, R. U. (2014). Influence of dietary zinc on semen traits and seminal plasma antioxidant enzymes and trace minerals of Beetal bucks. Reproduction in Domestic Animals, 48(6), 1004–1007. 10.1111/rda.12422 25263460

[rda14086-bib-0031] Roche, J. R. , Friggens, N. C. , Kay, J. K. , Fisher, M. W. , Stafford, K. J. , & Berry, D. P. (2009). Body condition score and its association with dairy cow productivity, health and welfare. Journal of Dairy Science, 92, 5769–5801. 19923585 10.3168/jds.2009-2431

[rda14086-bib-0032] Sampath, K. , Praveen, U. , & Kharaiah, M. C. (2004). Effect of strategic supplementation of finger millet straw on milk yield in crossbred cows on farm trial. Indian Journal of Dairy Science, 57(3), 192–197.

[rda14086-bib-0033] Saqib, M. N. , Qureshi, M. S. , & Khan, R. U. (2018). Changes in post‐partum metabolites and resumption of ovarian cyclicity in primiparous and multiparous dairy cows. Applied Biological Chemistry, 61, 107–111. 10.1007/s13765-017-0331-7

[rda14086-bib-0034] Shah, M. , Qureshi, M. S. , Khan, R. U. , Mobashar, M. , Khalique, M. A. , Khattak, I. , Tariq, A. , Ahmad, I. , & Naz, S. (2019). Semen quality of bulls as influenced by breed, body condition score and ascorbic acid under heat stress. Pakistan Journal of Zoology, 51, 1699–1703. 10.17582/journal.pjz/2019.51.5.1699.1703

[rda14086-bib-0035] SPSS (2004). User's guide, version 13.0. SPSS Inc.

[rda14086-bib-0036] Ullah, H. , Khan, R. U. , Mobashar, M. , Ahmad, S. , Sajid, A. , Khan, N. U. , Usman, T. , Khattak, I. , & Khan, H. (2019). Effect of yeast based selenium on blood progesterone, metabolites and milk yield in Achai dairy cows. Italian Journal of Animal Science, 18, 1445–1450. 10.1080/1828051X.2019.1683475

[rda14086-bib-0037] Ullah, H. , Khan, R. U. , Tufarelli, V. , & Laudadio, V. (2020). Selenium: an essential micronutrient for sustainable dairy cows production. Sustainability, 12(24), 10693. 10.3390/su122410693

[rda14086-bib-0038] Ullah, H. , Rahman, A. , Khan, R. U. , Ahmad, S. , Tariq, A. , & Naz, S. (2019). Breed, sex and body condition score impacted the meat quality of Waziri and Mazai sheep. Pakistan Journal of Zoology, 51, 1705–1710.

[rda14086-bib-0039] Zhao, W. , Chen, X. , Xiao, J. , Chen, X. H. , Zhang, X. F. , Wang, T. , Zhen, Y. G. , & Qin, G. X. (2019). Prepartum body condition score affects milk yield, lipid metabolism, and oxidation status of Holstein cows. Asian‐Australasian Journal of Animal Sciences, 32(12), 1889. 10.5713/ajas.18.0817 PMC6819678 31010972

